# Extracellular Vesicles and Their Role in Osteogenesis

**DOI:** 10.3390/bioengineering13091086

**Published:** 2026-09-19

**Authors:** Marcus Jäger, Andrea Sowislok

**Affiliations:** 1 Orthopedics and Trauma Surgery, University of Duisburg, 45147 Essen, Germany; andrea.sowislok@uni-due.de; 2 Department of Orthopedics, Trauma and Reconstructive Surgery, St. Marien Hospital Mülheim a.d. Ruhr, 45468 Mülheim, Germany; 3 Department of Orthopedics, Trauma and Reconstructive Surgery, Philippus Stift, 45345 Essen, Germany

**Keywords:** extracellular vesicles, exosomes, bone regeneration, osteogenesis, angiogenesis, bone tissue engineering

## Abstract

Extracellular vesicles (EVs) have emerged as promising cell-free therapeutic agents for bone regeneration due to their ability to modulate osteogenesis, angiogenesis, and immune responses. This review summarizes the current knowledge on EV biology, including their classification, biogenesis, and molecular cargo, with a particular focus on their role in bone healing. EVs derived from mesenchymal stromal cells and other cell types promote osteoblast differentiation, inhibit osteoclast activity, enhance vascularization, and modulate inflammation through the transfer of bioactive proteins and non-coding RNAs. Recent advances in EV engineering, biomaterial-assisted delivery, and isolation techniques are discussed, together with their translational potential for the treatment of critical bone defects and avascular osteonecrosis. Although preclinical evidence is highly encouraging, clinical evidence remains limited, particularly in orthopedics, and clinical translation is challenged by EV heterogeneity, lack of dose standardization, limited understanding of biodistribution, scalable manufacturing, and regulatory requirements. Overall, EV-based therapies represent a promising future strategy for regenerative orthopedics and bone tissue engineering.

## 1. Introduction

Critical bone defects and impaired bone healing remain a major challenge for orthopedic and trauma surgeons and affect people of all ages. These conditions also include avascular osteonecrosis (AVN), which, due to its progressive nature, leads to joint destruction. Since currently available natural and synthetic bone substitutes often lack sufficient osteoinductive capacity, significant efforts have been made in both research and industry to develop suitable biological agents that possess osteoinductive properties and are simultaneously safe and reliable to use. In this context, extracellular vesicles (EVs) have gained increasing attention as potential cell-free therapeutic agents for bone regeneration.

**Figure 1:**
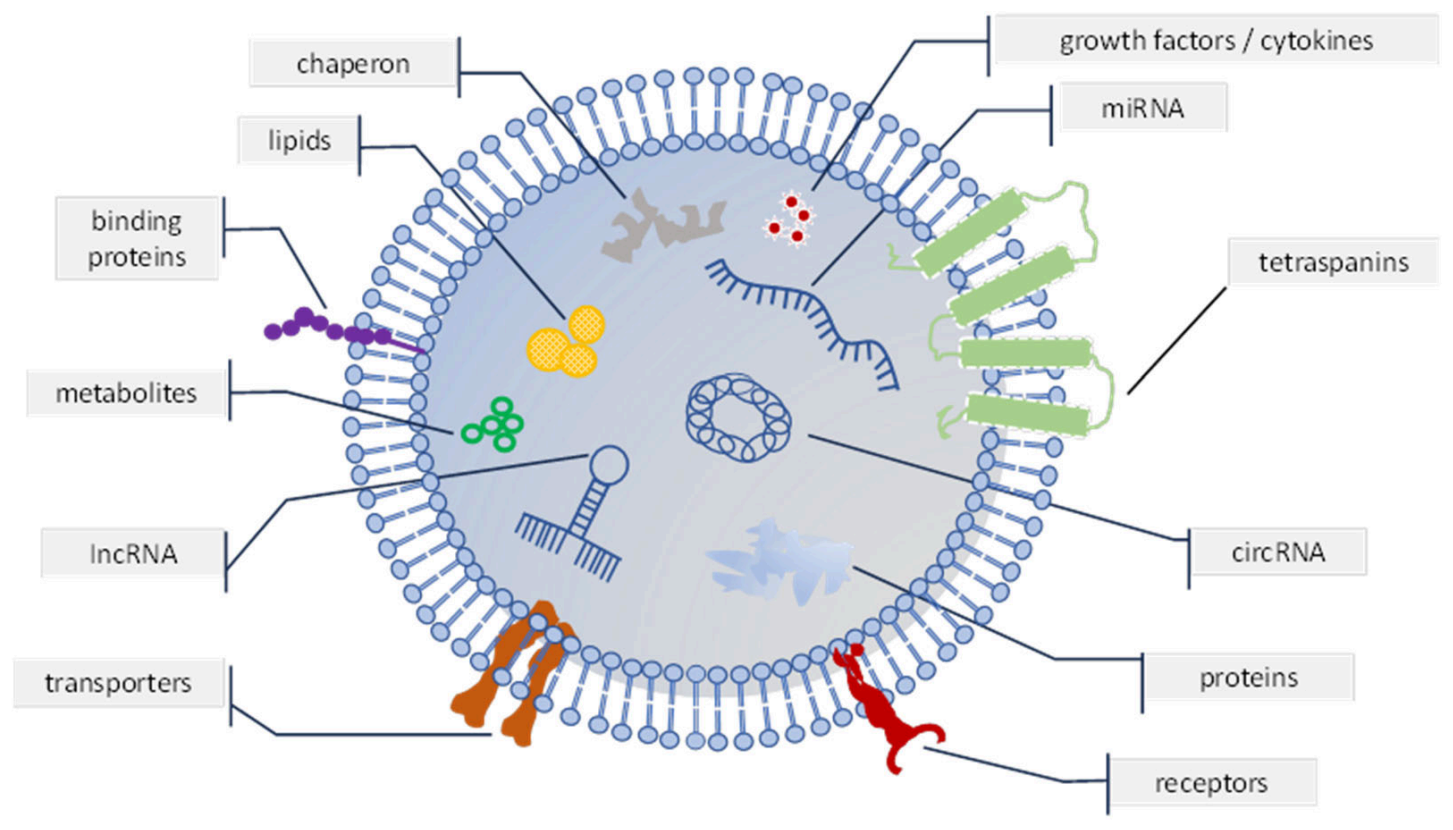
Extracellular vesicles generally contain a diverse range of RNA species, proteins, lipids and other bioactive molecules. Their membrane is enriched in functional proteins, including tetraspanins (e.g., CD9, CD63, CD81, and CD82), receptors (e.g., MHC class I/II, FasL, EGFR, PDGF, and VEGF), transporters (e.g., ABC and SLC transporters), and adhesion and binding proteins such as integrins and ICAMs. The intravesicular cargo comprises various nucleic acids, including mRNA, miRNA, and lncRNA; lipids such as cholesterol, sphingolipids, and ceramide; and proteins, including enzymes, cytokines, chaperones, heat shock proteins (e.g., HSP70 and HSP90), Rab GTPases, and cytoskeletal proteins such as actin, tubulin, vimentin, cofilin, profilin, and talin. EVs may also contain nucleotides, metabolites, and other bioactive molecules, which can contribute to intercellular communication and signal transduction. The major histocompatibility complex (MHC) is also commonly detected in EVs [2].

Extracellular vesicles (EVs) are of cellular origin and are enclosed by a lipid bilayer. They constitute a heterogeneous group that contains bioactive molecules derived from the cytosol (Figure 1). As mediators, these molecules play an important role in numerous physiological and pathological conditions [1]. They can act as carriers between cells but can also bind to the extracellular matrix. Depending on the EV population, particle size generally ranges from approximately 30 to 1000 nm (density: 1.06–1.21 g/mL), whereas apoptotic bodies can be considerably larger.

The classification systems and definitions of EV subtypes published in the scientific literature are used in a relatively inconsistent manner compared to other fields of cell biology (e.g., autophagic EVs, matrix EVs, stressed EVs) [3,4]. Therefore, international initiatives such as the Extracellular RNA Communication Consortium [5,6,7,8] and the International Society for Extracellular Vesicles [7,9] aim to establish standardized nomenclature and reporting criteria [10].

Unlike other forms of cell-to-cell communication, such as hormones, growth factors, cytokines, or direct cell-to-cell contacts, EV-mediated communication involves complex mechanisms that are still incompletely understood. However, due to rapid advances in this field [11,12,13], the first clinical applications of EVs are now emerging. For example, EVs were used to treat malignant melanoma prior to 2005, and the safety of this cell-free preparation was demonstrated (phase I clinical trial) [14].

In recent years, there has been growing evidence that EVs play an important role in bone formation and healing and may become a promising treatment for bone healing disorders, critical bone defects, and osteonecrosis [15,16,17]. Importantly, their effects are highly dependent on their cellular origin and molecular cargo. In osteonecrosis, EVs may therefore exert a dual role, either promoting regenerative processes or contributing to disease progression [18]. Among other things, EVs enhance the homing of osteoblasts to areas of bone defect and shorten the time required for bone mineralization [19]. EVs bind to proteins in the bone matrix (collagen I, fibronectin) and promote the differentiation of osteoblasts in vitro and in vivo [20]. The therapeutic use of EVs offers several advantages over cell therapies, including a comparably high level of biological safety and stability. Furthermore, EVs can be isolated relatively easily from tissue obtained during surgical procedures on the musculoskeletal system. For example, tissue fragments released during musculoskeletal, aseptic bone surgeries (surgical site released tissue) not only exhibit high in vitro osteogenic potential [21,22,23] and have already been successfully used clinically for the bioactivation of ceramic scaffolds [24,25,26], but also allow for the extraction of specific EVs under culture conditions [27]. These properties have stimulated increasing interest in the potential clinical application of EVs.

Based on a comprehensive review of the current literature retrieved from MedLine, Scopus, and Google Scholar, covering publications from 2000 to 2026, this article summarizes the biological functions of EVs relevant to bone regeneration, discusses recent advances in EV engineering and biomaterial-assisted delivery, and critically evaluates their translational potential for the treatment of bone healing disorders. Searches focused on EVs, bone regeneration, osteogenesis, angiogenesis, osteonecrosis, EV engineering, therapeutic cargo, and biomaterial-assisted delivery, with additional relevant studies identified from reference lists.

## 2. Biology of Extracellular Vesicles

Extracellular vesicles (EVs) comprise a heterogeneous population of membrane-enclosed particles that differ in size, biogenesis, molecular composition, and biological function. Although the International Society for Extracellular Vesicles (ISEV) recommends using the generic term “extracellular vesicles” unless their biogenesis has been experimentally demonstrated, the traditional classification into exosomes, microvesicles, migrasomes and apoptotic bodies remains widely used in the literature and is therefore presented here as a conceptual framework rather than as a definitive classification (Table 1).

**Figure 2:**
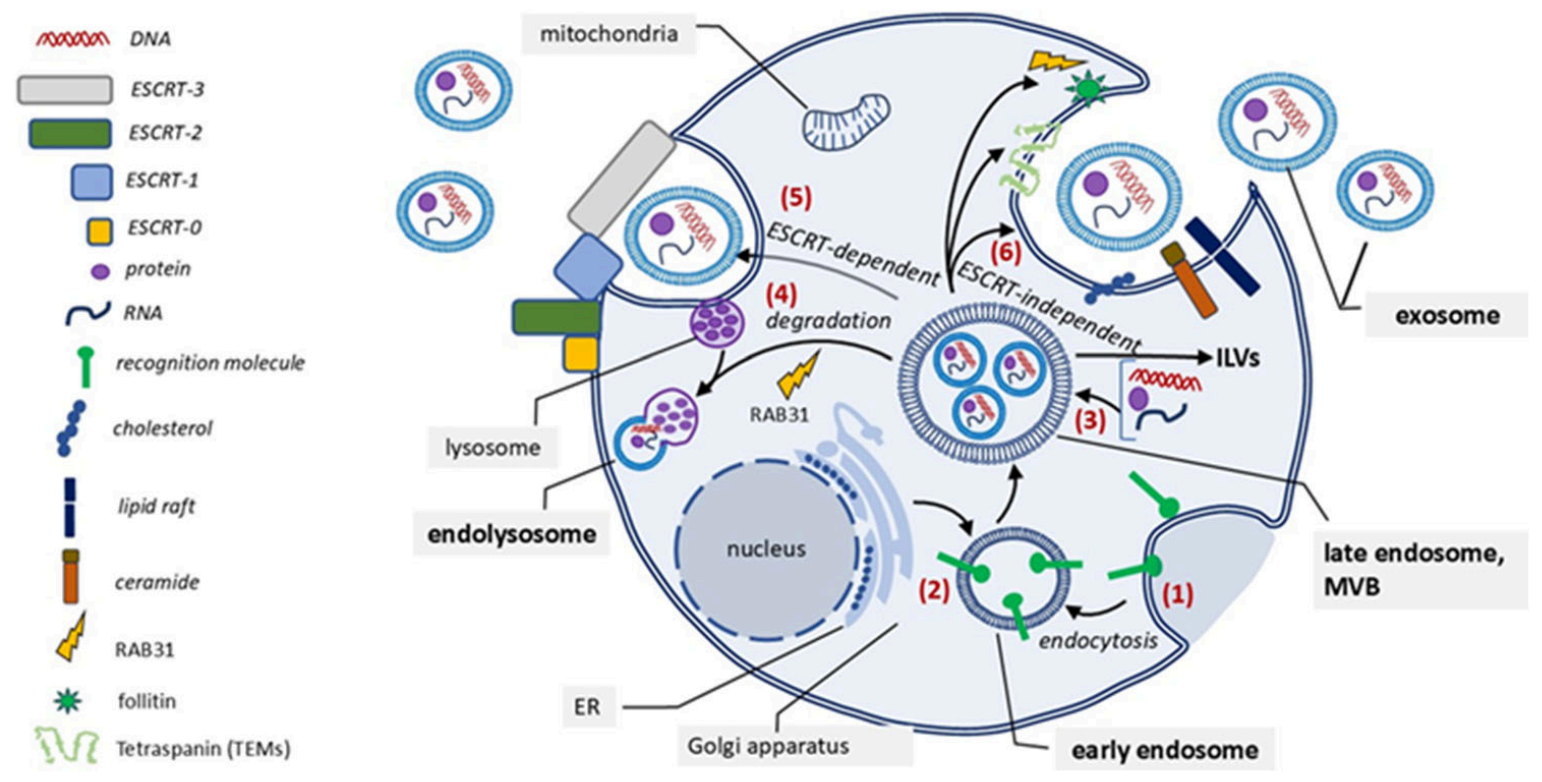
The biogenesis of exosomes starts with endocytosis, where plasma membrane invagination forms a cup-shaped structure followed by internal budding of the cell membrane (1) and the inclusion of additional bioactive molecules within the cytosol. This internal budding of the cell membrane, together with its cell surface proteins and extracellular components, e.g., bioactive molecules, results in early endosome formation (2). Within the endosomal lumen, smaller multivesicular bodies (MVBs) are formed (3), containing intraluminal vesicles (ILVs). The further fate of MVBs includes several pathways. Some MVBs undergo degradation by fusion with lysosomes (4), forming endolysosomes. Other MVBs fuse with the plasma membrane (5), resulting in the release of ILVs into the extracellular space, where they are termed exosomes (6). ILV formation can involve the ESCRT machinery or ESCRT-independent mechanisms. The protein Rab31 has been reported to regulate MVB trafficking and lysosomal degradation, thereby influencing the release of EVs [30,31].

**Exosomes** are a well-described EV subtype, are enclosed by a lipid bilayer membrane, and are released by many cell types. They are derived from intraluminal vesicles (ILVs), which are formed within multivesicular bodies (MVBs). Upon fusion of MVBs with the plasma membrane, ILVs are released into the extracellular space and are then termed exosomes. They are found in plasma, urine, semen, saliva, and cerebrospinal fluid, as well as in bronchial secretions, breast milk, serum, amniotic fluid, synovial fluid, and bile, gastric juice, and tears. Functionally, exosomes serve to rid cells of unwanted material and thus contribute to cellular maintenance. However, they also play a role in cell-to-cell communication, stimulate the immune system by presenting antigens, stabilize the myelin sheath around nerve cells, and play an important role in the repair and regeneration of tissue defects. Exosome biogenesis occurs intracellularly through ESCRT-dependent pathways or alternative mechanisms, including ceramide-dependent budding, tetraspanin-enriched microdomains involving CD63, CD9, and CD81 that contribute to membrane curvature and cargo recruitment, and the Rab31/flotillin pathway. Figure 2 presents important steps in exosome biogenesis.

In contrast, **microvesicles** are generated by outward budding and subsequent fission of the cell membrane (an energy-dependent process). They typically contain cytoskeletal proteins such as actin, microtubules, kinesin, and myosin, as well as fusion molecules (SNAREs and other binding factors). Due to their formation, they also contain cellular membrane proteins, some of which are present in high concentrations (e.g., tetraspanins, integrins, and heat shock proteins). Glycan-binding proteins, in particular, appear to play an important role in the uptake by target cells. However, the uptake of microvesicles by target cells depends just as much on the cells’ biological state. For example, low temperatures reduce uptake.

**Migrasomes** form during cell migration, particularly at tips or intersections of retracting fibers. Due to multiple internal vacuoles, they have a pomegranate-like appearance. Migrasomes are involved in intercellular communication and carry growth factors, cyto- and chemokines. Furthermore, they support cells in secreting damaged proteins or organelles (such as mitochondria) and maintain internal homeostasis [2,32,33]. There is evidence that M2 macrophage-derived migrasomes promote bone fracture healing by orchestrating stem cell homing and osteogenic differentiation in BM-MSCs [34].

**Apoptotic bodies** are released into the extracellular space by dying cells and have a diameter ranging from 50 nm to 5000 nm, with most apoptotic bodies being larger. They contain relatively high amounts of the cellular cytoskeleton, including intact organelles and chromatin, since the cell contracts as it releases them [35]. Some data suggest that apoptotic extracellular vesicles may also orchestrate the bone healing niche, e.g., by aggregation and macrophage interactions [36].

## 3. Role of EVs in Bone Regeneration

### 3.1. Promotion of Osteogenesis

Due to their biological properties, EVs are increasingly being investigated as potential candidates for the treatment of tissue defects [37]. EVs derived from human bone marrow-derived mesenchymal stromal cells (BM-MSCs) contain several osteogenic miRNAs, including miR-196a, miR-27a, and miR-206. For example, miR-196a promotes the expression of ALP, OCN, osteopontin, and RUNX-2 and enhances bone healing in experimental calvarial defects [38]. While additional osteogenic miRNAs are upregulated in the target cells of the microenvironment surrounding bone defects (miR-146a-5p, miR-503-5p, miR-483-3p, miR-129-5p), anti-osteogenic miRNAs are downregulated. These effects have been associated with activation of the PI3K/Akt and MAPK signaling pathways, thereby promoting bone mineralization [39].

In addition to miRNAs, EVs transport other functional RNA species, including long non-coding RNAs, which can regulate the expression of genes involved in bone formation [40]. EV-associated mRNAs may also be translated into functional proteins in recipient cells, while protein cargo can directly modulate cellular signaling. Recent studies have further demonstrated the capacity of EVs to efficiently deliver functional proteins to recipient cells, highlighting their potential as natural carriers of therapeutic biomolecules [41]. EVs can also activate additional intracellular signaling cascades involved in osteogenesis and the healing of critically large bone defects in rats (BMP/Smad, Wnt/β-catenin, tensin homolog/PI3K/Akt, Hippo) [42].

The biological activity of EVs is not restricted to a single cargo molecule or EV subtype. EVs released by mesenchymal stromal cells (MSC-EVs), adipocytes, or platelets also play a significant role in bone healing. For example, iPS-MSC exosomes promote both osteogenesis and angiogenesis through their internal miRNAs [43] even in the presence of the bone substitute β-TCP [44]. Similarly, exosomes derived from human adipose-derived stromal cells (ADSCs) promoted bone healing in rats after immobilization on polydopamine-coated PLGA scaffolds [45]. Several studies have shown that exosomal miRNAs increase VEGF expression in MSCs, thereby promoting vascularization. The contribution of protein cargo is further supported by proteomic analyses of EVs derived from osteoblasts and MC3T3 cells, which identified approximately 786 and 172 proteins, respectively, associated with osteogenic processes [46,47].

In summary, numerous studies demonstrate that osteogenesis is mediated by a heterogeneous group of EVs derived from different cell types. Their effects are largely attributed to the transfer of diverse bioactive cargo, including miRNAs, mRNAs, and proteins, into the microenvironment of the bone defect.

### 3.2. Regulation of Osteoclast Activity

In addition, EVs modulate the balance between osteogenesis and osteoclastogenesis by inhibiting osteoclast differentiation through several mechanisms [17,48,49]. These include:

- Downregulation of miR-214 expression and inhibition of the NF-κB signaling pathway (EVs from prostate cells) [50].
- Inhibition of the expression of tartrate-resistant acid phosphatase, cathepsin K, and matrix metalloproteinase-9 [51].
- Inhibition of RANKL mRNA and protein expression (EVs from ADSCs) [52].
- Reduction in the bone resorption rate (EVs from endothelial cells) [53].

Conversely, EVs derived from osteoclasts are enriched in miRNAs that can negatively affect osteoblast activity. In particular, miR-214, miR-214-3p, and miR-23a-5p have been reported to inhibit RUNX2 activity, thereby impairing osteoblast function [54,55].

Under physiological conditions, EVs are exchanged between osteoblasts, osteocytes, and osteoclasts, as well as their precursor cells. Similarly, adipocytes, myoblasts, the endothelium, dendritic cells, and synovial fibroblasts secrete exosomes. Some EV populations can contribute to mineralization. Matrix vesicles represent a specialized EV population associated with the extracellular matrix and mineral deposition and should be distinguished from endosome-derived exosomes.

### 3.3. Promotion of Angiogenesis

In addition to bone formation (differentiation of osteoblasts, mineralization of the extracellular matrix) and the inhibition of bone resorption, numerous EVs promote angiogenesis, which is a prerequisite for bone healing [56,57,58]. Proangiogenic EVs have been identified at bony tendon insertions [59,60], in EVs derived from endothelial progenitor cells (miR-126), and in MSC-EVs (miR-21) [58,61]. The mechanisms involved here vary and include the downregulation of Sprouty homolog 2 expression, increased expression of VEGF, angiopoietin 1 and 2, collagen-1 synthesis, and activation of the Hippo signaling cascade [62,63]. Direct promotion of the migration of other cell types into the bone defect (chemotaxis) has also been described [64,65].

Physiologically, tissue hypoxia following fracture or surgery increases miR-126 expression in MSC-derived exosomes, thereby stimulating angiogenesis, proliferation, and migration of stromal cells [65,66,67]. Some studies in the scientific literature indicate that lithium-containing biomaterials also induce miR-130a expression in exosomes and activate the tensin homolog/Akt signaling pathway, which in turn stimulates angiogenesis in a bone defect [66].

In summary, specific EVs stimulate angiogenesis in bone defects and thereby contribute to healing.

### 3.4. Immunomodulatory Effects of EVs

Another effect of EVs is to modulate the immune response in bone defects, which are always accompanied by a local inflammatory response [68,69,70]. This includes the functional regulation of T and B cells, macrophages, and other cells through direct membrane fusion or indirectly through the transport of signaling molecules [71]. In vitro studies on MSC-EVs have shown that they inhibit both T-cell and B-cell responses and also suppress the release of proinflammatory factors (TNF-α, IL-1β) [72]. However, EVs can also modulate macrophage function as part of the innate immune response. For example, EVs have been reported to modulate macrophage activation, including a shift toward an M2-like, anti-inflammatory phenotype, accompanied by reduced IL-6 synthesis and modulation of the NF-κB signaling pathway [73,74,75,76]. Although the M1/M2 classification represents a simplified model of macrophage activation, a shift toward an M2-like phenotype has been associated with reduced local inflammation and enhanced tissue regeneration.

In particular, miR-451a, which is highly expressed in ADSC EVs, has been implicated in these effects [77,78], whereas human umbilical vein endothelial cell (HUVEC)-derived exosomes have been shown to have a T-cell-suppressive effect.

**Figure 3:**
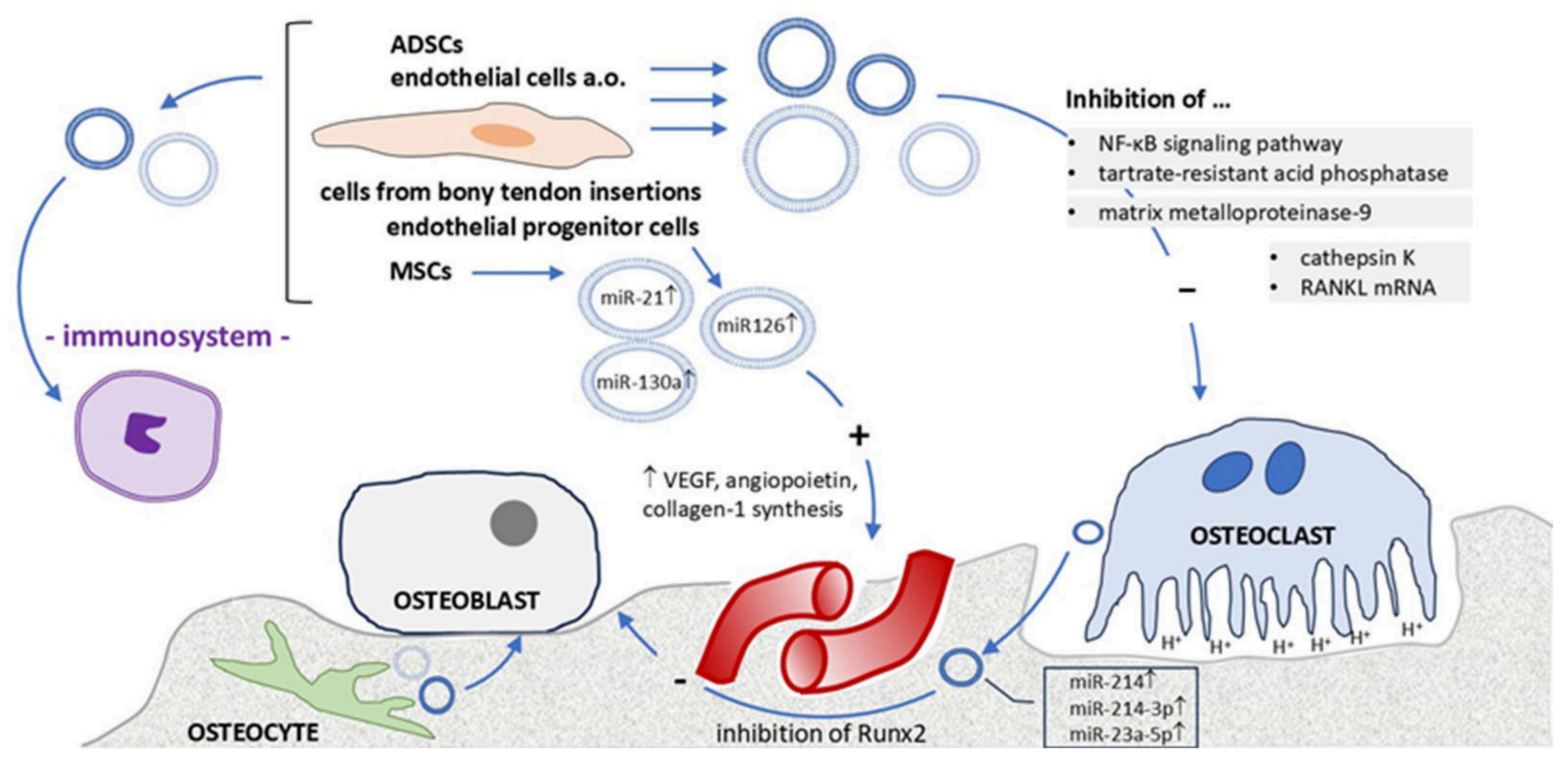
Dependent on source, cell type and condition, EVs can inhibit osteoclasts, promote angiogenesis and bone regeneration (osteoblast differentiation, mineralization). Furthermore, some EVs can potently modulate cell-dependent immune response. Exosomes contain a range of biomolecules that promote bone healing but can also accelerate resorption [79].

Collectively, EVs promote osteogenic differentiation, inhibit osteoclastogenesis, stimulate angiogenesis, and modulate the immune response, thereby creating a microenvironment that supports bone healing (Figure 3).

## 4. Pathophysiological Effects of EVs in Osteonecrosis of the Femoral Head

**Figure 4:**
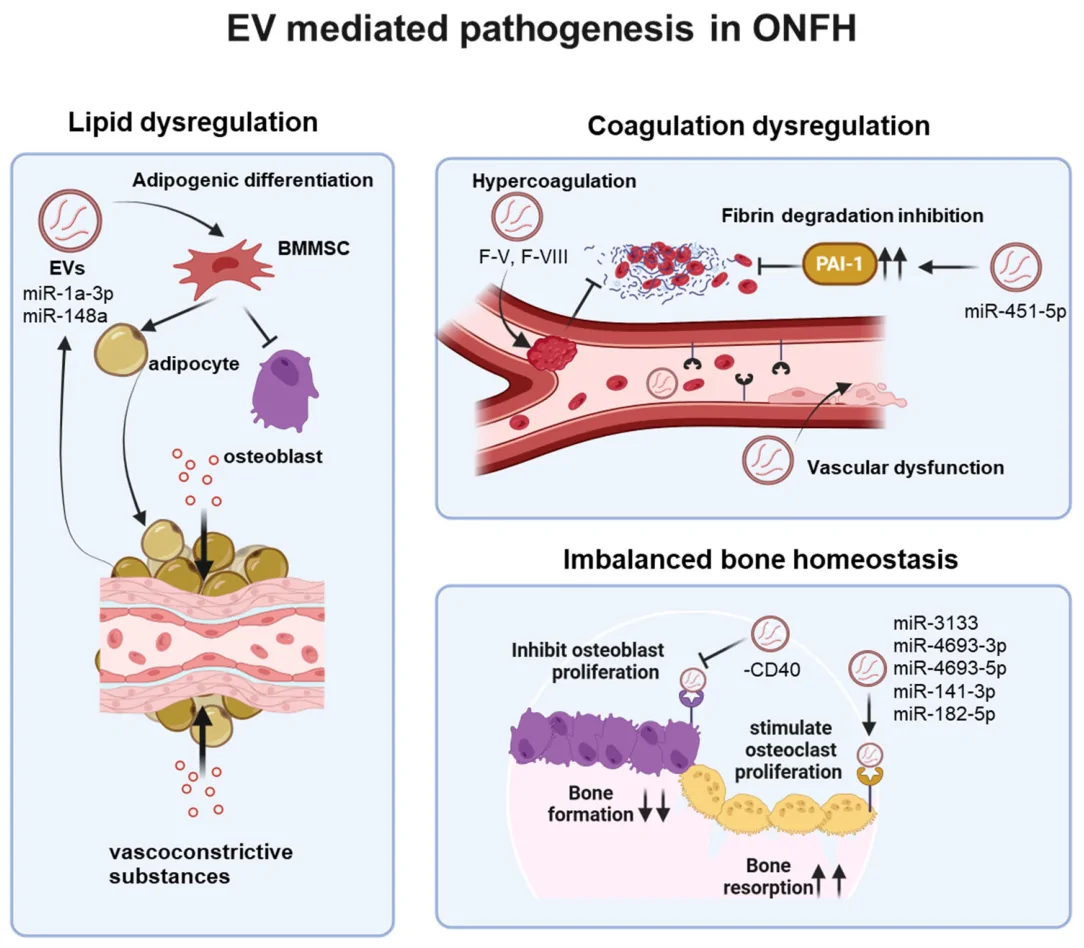
Schematic overview of the main EV-mediated mechanisms involved in the pathogenesis of osteonecrosis of the femoral head (ONFH), including adipogenic differentiation of bone marrow mesenchymal stromal cells (BM-MSCs), vascular dysfunction and alterations in osteoblast and osteoclast activity [18]. Created in BioRender.

EVs may play a dual role in osteonecrosis of the femoral head (ONFH). Depending on their cellular origin and molecular cargo, they may either contribute to disease progression or support tissue regeneration [18]. While their regenerative effects have been discussed above, this section focuses on their potential contribution to ONFH pathophysiology. A summary of the pathological processes is shown in Figure 4.

A key mechanism in ONFH is the imbalance between osteogenesis and adipogenesis in bone marrow mesenchymal stromal cells (BM-MSCs). In glucocorticoid-associated ONFH, macrophage-derived EVs enriched in miR-1a-3p have been reported to promote adipogenic differentiation and suppress osteogenesis, while adipocyte-derived EVs containing miR-148a may further enhance adipogenesis through the Wnt5a/Ror2 pathway [80,81]. Reduced miR-182-5p in steroid-induced ONFH has also been associated with enhanced MYD88/MAPK/NF-κB signaling and impaired RUNX2-mediated osteogenesis [82]. Increased marrow adiposity may subsequently contribute to deterioration of the local vascular and metabolic microenvironment.

The vascular component of ONFH may likewise be influenced by EVs. Increased platelet- and endothelial-derived microvesicles may promote coagulation, microvascular thrombosis, and inflammation, potentially further compromising the blood supply to the femoral head [83,84]. In addition, pathological EVs may enhance osteoclast activity and bone resorption, potentially contributing to progressive structural deterioration of the femoral head [85]. Together, these mechanisms may contribute to a vicious cycle of impaired osteogenesis, increased adipogenesis and bone resorption, inflammation, and vascular dysfunction.

## 5. Engineering and Modification of EVs for Bone Regeneration

**Figure 5:**
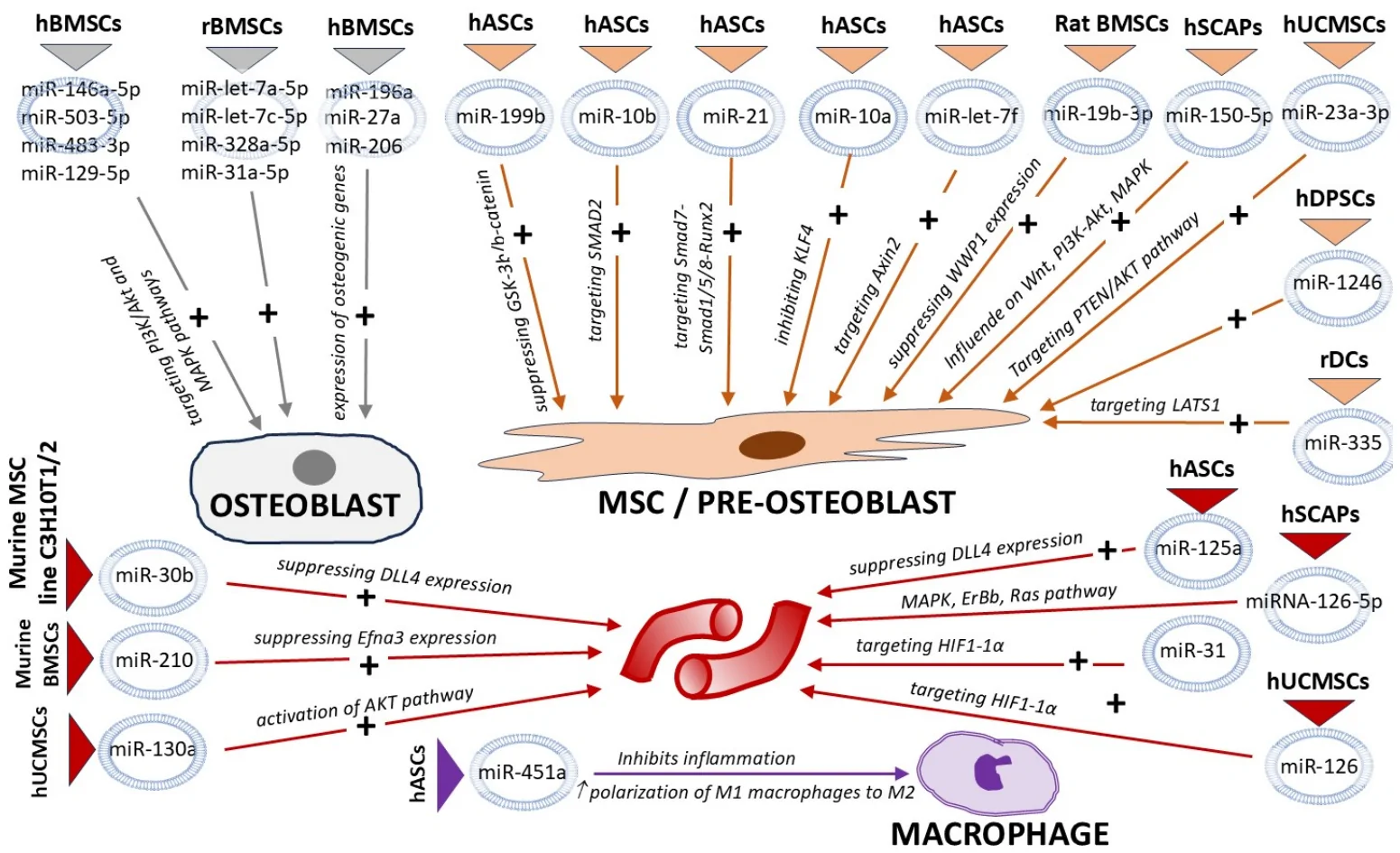
Typical parent cells (∇) and expressed miRNAs in EVs which are involved in bone regeneration. Most studies focus on MSCs, osteoblast precursors or angiogenic cells as target for EV cargo, whereas less knowledge exists of the detailed immunogenic influence of EVs on bone regeneration [38,39,40,65,67,78,98,117,118,119,120,121,122,123].

Most studies aimed at clinical application in the field of bone healing focus on EVs as cellular transport vehicles for miRNAs and proteins, while data examining the complex bone healing processes mediated by EVs in vivo are still limited. Some studies have focused on specific EV subtypes or chemically modified EVs to achieve higher concentrations of bioactive molecules at the target site and more targeted effects on recipient cells, thereby enhancing osteogenesis. In principle, EV modification can be achieved through endogenous or exogenous approaches. For bone regeneration, preconditioning through co-cultivation techniques appears promising. In addition to endogenously modified EVs, they can also be specifically produced exogenously for bone regeneration (Figure 5). EV engineering remains an active field of research [12,86,87,88,89]. Established EV modification techniques and their specific limitations are summarized in Table 2.

Recent preclinical studies have demonstrated the therapeutic potential of cargo-specific engineered EVs for bone regeneration. mRNA-engineered EVs carrying BMP-2 mRNA have been shown to enhance bone regeneration when incorporated into a GelMA hydrogel in a rat calvarial defect model [112]. Similarly, EVs enriched with VEGF-A and BMP-2 mRNAs promoted both vascularization and bone regeneration in critical-size rat femoral defects when delivered within a PEGS-A hydrogel [113]. Protein-based engineered EVs represent another promising approach. Intraluminally loaded BMP-2 showed increased protection against proteolysis and Noggin-mediated inhibition while retaining osteogenic activity [114]. Furthermore, EVs containing both BMP-2 and BMP-7 enhanced bone formation in a rat calvarial defect model, with effects comparable to rhBMP-2 [115]. These findings highlight the potential of cargo-specific EV engineering for localized delivery of osteogenic and angiogenic factors. Representative engineered EV strategies and their therapeutic applications are summarized in Table 3.

Despite their numerous advantages [13], none of the techniques mentioned are suitable for the intraoperative collection of SSRT-EVs for bone regeneration for various reasons (technical and logistical challenges, time required, costs, patient risks). Likewise, preconditioning the source cells to enhance performance as described by some authors is not feasible in this setting [116].

### Biomaterial-Assisted EV Delivery

Another challenge involves the local retention and concentration of EVs. Without binding to a scaffold, EVs rapidly diffuse from the desired target site into adjacent tissue compartments and ultimately into the lymphatic system and the bloodstream. For example, BM-MSC EVs have already been successfully bound to β-TCP [94], ethylene glycol methacrylate-chitosan hydrogels [124] and bioactive glasses [98]. The surface properties of the biomaterial (surface area, pore size, electrostatic properties, etc.) are critical for EV binding to bone substitutes [29]. In addition to efficient binding and an appropriate loading capacity, it is also necessary to ensure the controlled release of EVs within the target tissue following application. In addition to the efficacy of the desired effect (pharmacodynamics), the distribution and bioelimination of EVs in pathological conditions (pharmacokinetics) in vivo in bone defects remain unclear.

**Figure 6:**
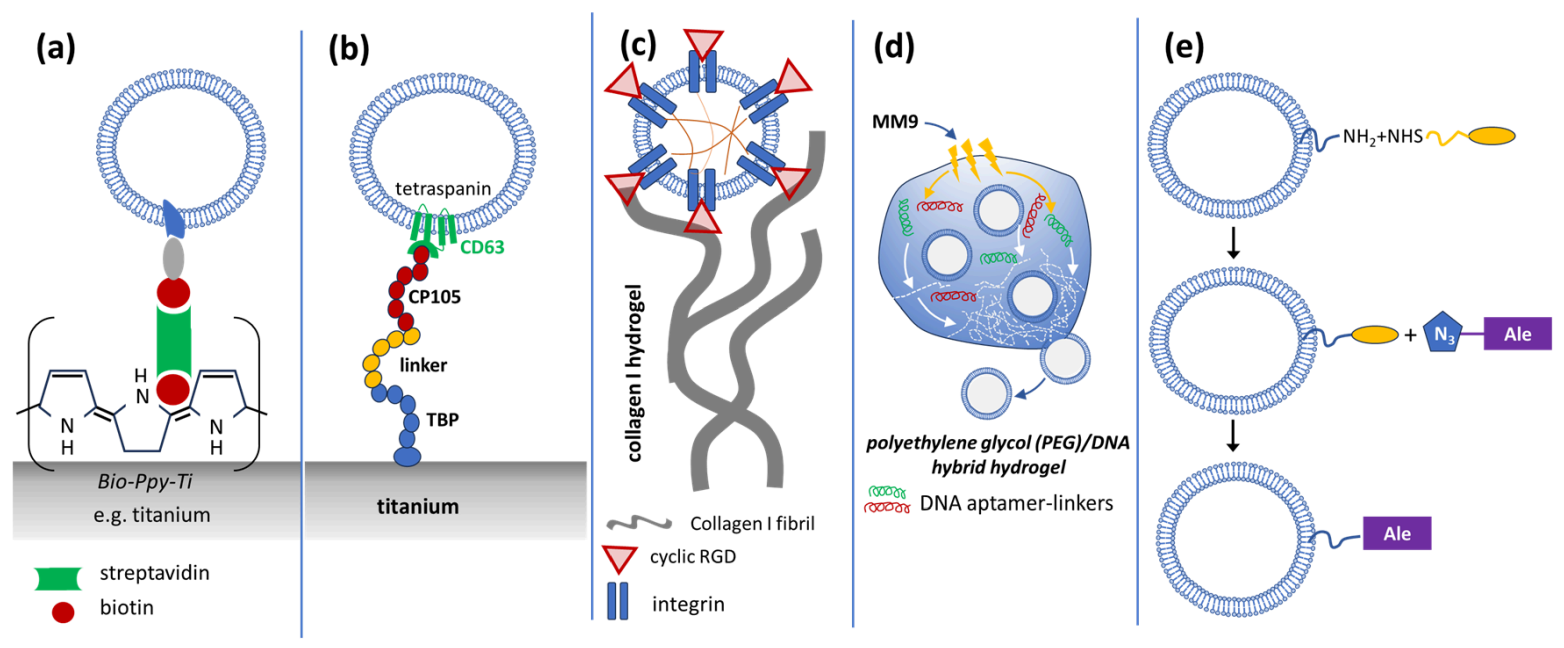
Biomaterial-EV interactions. Various techniques exist for binding EVs to biomaterial surfaces including the (**a**) avidin-biotin system [118], (**b**) tetraspanin CD63-TBP-CP05 fusion, (**c**) RGD-integrin interactions [138] (e.g., within collagen hydrogels) or (**d**) EV cross-linking within hydrogel pores (encapsulation). In the latter approach, matrix metalloproteinase-9 releases the embedded DNA aptamer-linker followed by disintegration of the DNA hydrogel and subsequent release of EVs [65]. (**e**) Other binding techniques follow click chemistry as demonstrated for alkynyl-group-modified EV (EVs-DBCO) conjugation with a modified Ale-N3 group [139].

In this context, simple physical adsorption of EVs onto biomaterial scaffolds appears insufficient, as it does not ensure controlled and sustained release of EVs. Polymers such as polyethylene glycol (PEG)/DNA hybrid hydrogels may overcome some of these limitations by improving long-term preservation, protecting EV bioactivity, and enabling controlled release at bone defect sites. Also, other injectable gels based on gelatin, hyaluronic acid, chitosan, silk PLGA, Pluronic127, or PEGDA are potential candidates for chemical cross-linking of EVs [125]. Another strategy for binding EVs onto scaffolds is based on biomaterial surface coating by RGD-peptides, a conserved integrin recognition motif. Here, especially EVs with integrin-rich membranes might adhere [126,127]. Figure 6 shows characteristic techniques conjugating EVs onto biomaterials. Other approaches enabling targeted delivery of therapeutic cargoes to osteoblasts and other cell types are based on multivalent electrostatic and hydrophobic interactions [128,129,130].

Our research group was not only able to isolate EVs from SSRT [26] but also found high concentrations of VEGF in this tissue [22]. Other research groups have also demonstrated the critical role of VEGF in bone healing in the context of EV-induced expression [30,86,131]. But not only the expression of growth factors, cytokines, or the release of EVs are influenced by biomaterials. Biomaterials have a strong impact on the microenvironment by interacting with local immune cells. Here, an enhanced M2 polarization is described for titanium, biphasic calcium phosphate ceramics, and collagen/sulfated HA biomaterials [132,133,134]. M2-like macrophages can release EVs containing cargo such as miR-23a-3p, which may further reduce local inflammation and support tissue regeneration [135,136,137].

## 6. Isolation of EVs

**Figure 7:**
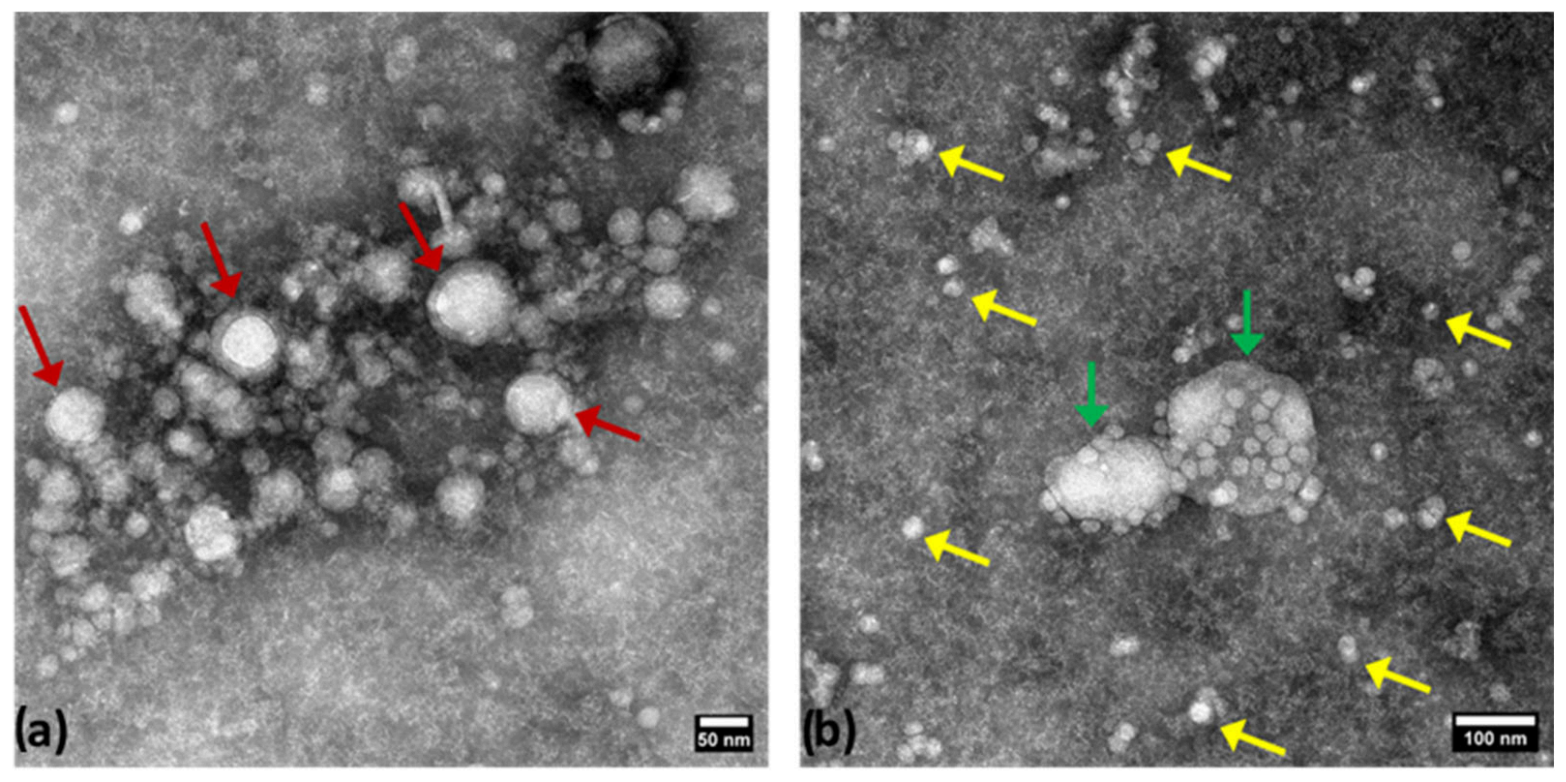
Representative transmission electron microscopy (TEM) images of EV preparations derived from human BM-MSCs following phosphotungstic acid negative staining. (**a**) Small EVs (**→**), imaged at a magnification of ×60,000, measuring 59–93 nm in diameter, with a clearly visible lipid bilayer membrane of approximately 8–10 nm thickness. (**b**) Larger EVs (**→**) imaged at ×50,000, ranging from 60 to 200 nm in diameter. Cholesterol particles (**→**) originating from the sample background are highlighted in yellow. The TEM morphology and size of these particles do not by themselves permit assignment to a specific EV subtype. (Own data).

**Figure 8:**
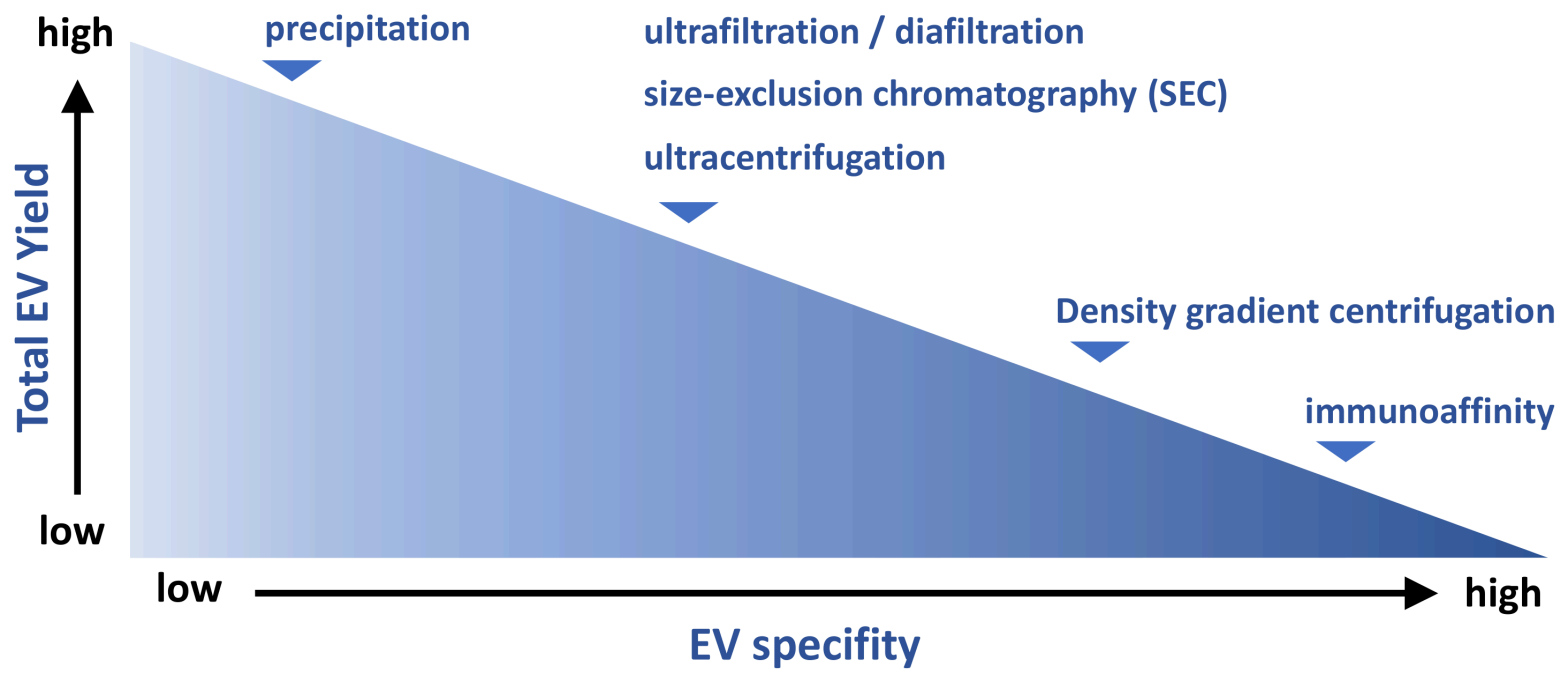
Relationship between the quantity and specificity of isolated EVs and the technique used, modified from https://www.novusbio.com/research-topics/cell-biology/exosome-isolation-and-detection (accessed on 16 June 2026).

Given that a single cell type can release heterogeneous EV populations, the controlled production of a defined EV population for clinical use remains challenging [34]. This inherent heterogeneity complicates the definition of a reproducible therapeutic product and makes dose standardization difficult, as EV preparations may differ in particle number, size distribution, molecular cargo, and biological activity. For one-stage, autologous applications, EV isolation must be rapid, reproducible, and compatible with intraoperative use [140]. Representative TEM images of EV preparations are shown in Figure 7. Common approaches include centrifugation, ultrafiltration, precipitation, size-exclusion chromatography, and affinity-based methods [141,142,143,144,145,146,147,148,149,150] (Table 4), but no single method currently combines optimal yield, purity, reproducibility, scalability, and processing speed. An overview of the relationship between EV quantity and specificity for different isolation techniques is shown in Figure 8.

In addition, the biodistribution, tissue retention, and clearance of EVs after administration remain insufficiently characterized and may depend on EV source, formulation, administration route, and disease context. Standardized isolation workflows that preserve EV bioactivity and therapeutic cargo will therefore be essential for clinical translation, particularly for engineered EV therapeutics.

## 7. Preclinical Application and Clinical Trials of EVs for Bone Regeneration

Most preclinical studies support the therapeutic potential of EVs for promoting osteogenesis. In particular, bone marrow-derived EVs may contribute to bone regeneration by activating the Wnt/β-catenin pathway in recipient BM-MSCs [151] among other mechanisms. In a mouse femoral defect model, MSC-derived, BMP-2-enriched EVs were combined with a collagen hydrogel and injected, showing a high biocompatibility and increased bone healing [152]. Comparable results were noted in a femoral fracture model in rats. Here BM-MSC exosomes promoted osteogenesis as well as angiogenesis [64]. These findings are consistent with studies by other groups demonstrating EV-mediated enhancement of bone healing even under pathologic conditions such as osteoporosis or diabetes [153,154,155]. However, in contrast to a large number of inuncle vitro and animal studies, the translation of EV-based approaches into human clinical trials remains challenging. Clinical evidence remains limited, particularly in the field of bone regeneration and orthopedics. Important translational barriers include the intrinsic heterogeneity of EV preparations, the lack of standardized dosing and potency assays, insufficient knowledge of EV biodistribution, tissue retention and clearance, as well as challenges related to scalable and reproducible manufacturing, quality control, and regulatory requirements [156]. These factors complicate the comparison of studies and the definition of consistent therapeutic products and treatment protocols. To date, about 80% of all EV-related clinical trials have examined EVs as diagnostics or as companion diagnostics. Over the last 15 years, first therapeutic clinical trials have also emerged, with the majority focusing on COVID-19, including acute and long COVID, and acute respiratory distress syndrome (ARDS) (intravenous > inhalation > topical). Here, bone marrow-derived MSC-EVs were mostly utilized. In the past, there was also a trend toward using native EVs in contrast to engineered EVs [157]. The number of clinical EV trials in orthopedics is very low, with available studies largely limited to phase I trials. For the treatment of low back pain, exosomes have been investigated for intradiscal administration in combination with platelet-rich plasma (PRP) (#NCT04849429/2022). Another study is investigating the safety of BM-MSC-derived EVs in deep grade II skin burns (#NCT05078385/2024). Moreover, individual healing attempts are not captured in major clinical trial registries and are not reported in the literature.

EV heterogeneity represents a challenge and an opportunity: while it may complicate the development of consistent and reproducible therapeutic products, it may also provide opportunities to identify or engineer EV subpopulations with enhanced osteogenic activity [158]. Standardized EV characterization, selection of functionally defined EV subpopulations, and improved potency assays may help address these challenges. In this context, EV engineering and hybrid delivery strategies may help improve cargo loading, tissue targeting, scalability, and therapeutic efficacy, thereby supporting the translation of EV-based osteogenic therapies into clinical applications [159].

Although the available preclinical evidence may indicate a shift in orthopedics from a focus on biological mechanism towards bioengineering-based therapeutic applications, it remains unclear whether EVs can overcome current translational bottlenecks and become a clinically relevant treatment option for bone healing disorders. Addressing these challenges will require interdisciplinary collaboration including smart biomaterials for EV delivery, nanotherapeutic approaches, scalable manufacturing, and standardized characterization and potency assays.

## 8. Conclusions and Future Perspectives

Extracellular vesicles represent a promising cell-free therapeutic approach for bone regeneration by promoting osteogenesis, angiogenesis, and immunomodulation. In ONFH, EVs may exert a dual role, supporting tissue repair or contributing to disease progression depending on their cellular origin and molecular cargo. Despite considerable progress in EV research, clinical evidence remains limited, and major challenges include EV heterogeneity, lack of standardized dosing, limited characterization of EV biodistribution, tissue retention, and clearance, as well as scalable and reproducible manufacturing, quality control, and regulatory approval before EV-based therapies can enter routine clinical practice. Addressing these challenges through interdisciplinary research will be essential to establish EV-based therapeutics as a viable treatment option for critical bone defects and impaired bone healing.

## Figures and Tables

**Table 1:** Commonly described EV subtypes and their reported size ranges, markers, and biogenesis. Size, morphology, molecular markers, or isolation methods alone are not sufficient to definitively assign EV subtype identity; subtype designation requires appropriate evidence of biogenesis and/or additional characterization [25,28,29].

EV Subtype	Typical Reported Size (Shape)	Commonly Reported Markers	Biogenesis/Origin	
Exosomes	~30–150 nm (mostly round)	Tetraspanins, Alix, TSG101, PDCD6IP, flotillin, MFGE8, CD63, CD82, CD9	Endolysosomal pathway; release of ILVs from MVBs	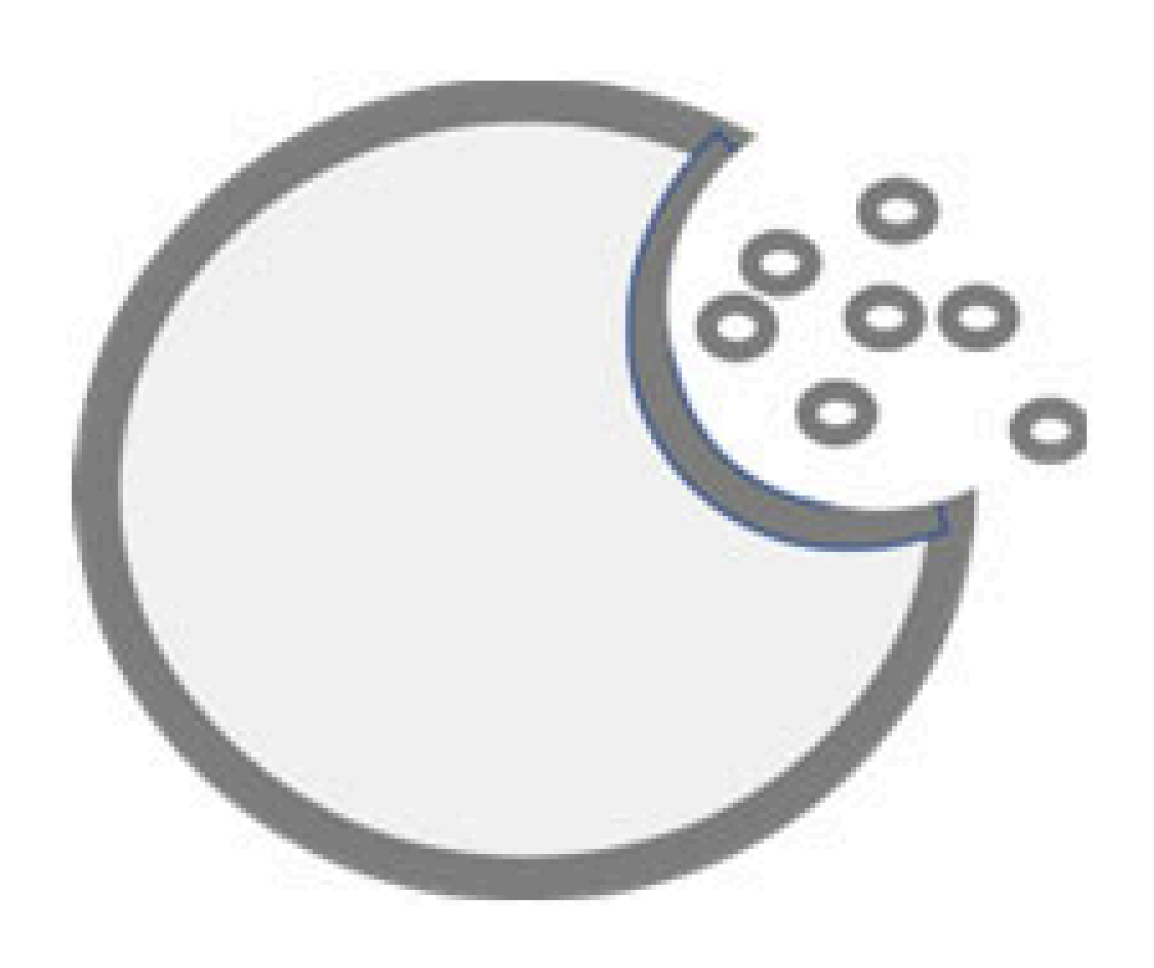
Microvesicles	~50–1000 nm (irregular)	Integrins, MMPs, selectins, CD40	Outward budding of the plasma membrane	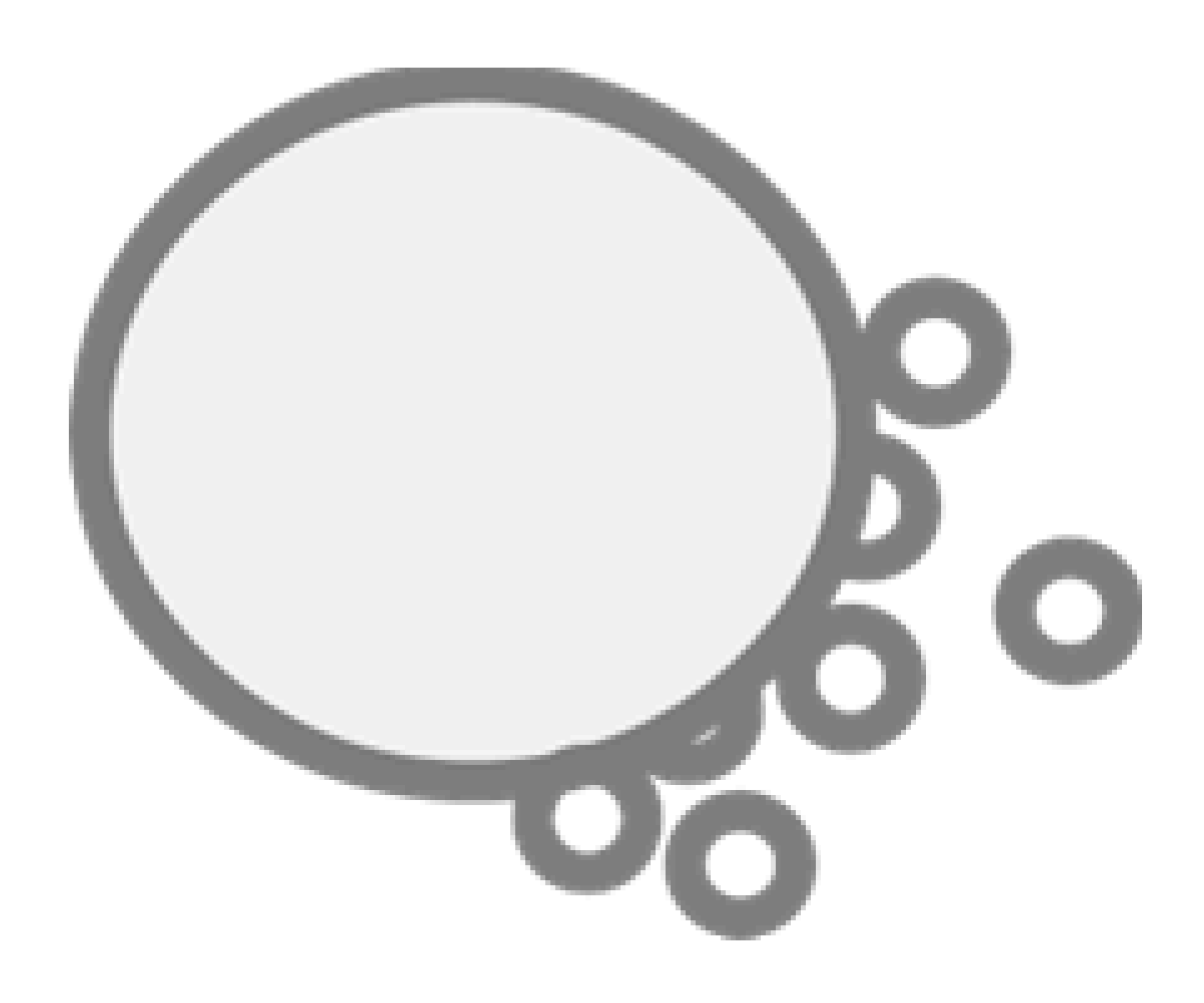
Migrasomes	~500–3000 nm	Tetraspanin-enriched macrodomains, TSPAN4, integrin, phosphatidylserine	Form at tips and intersections of retracting fibers	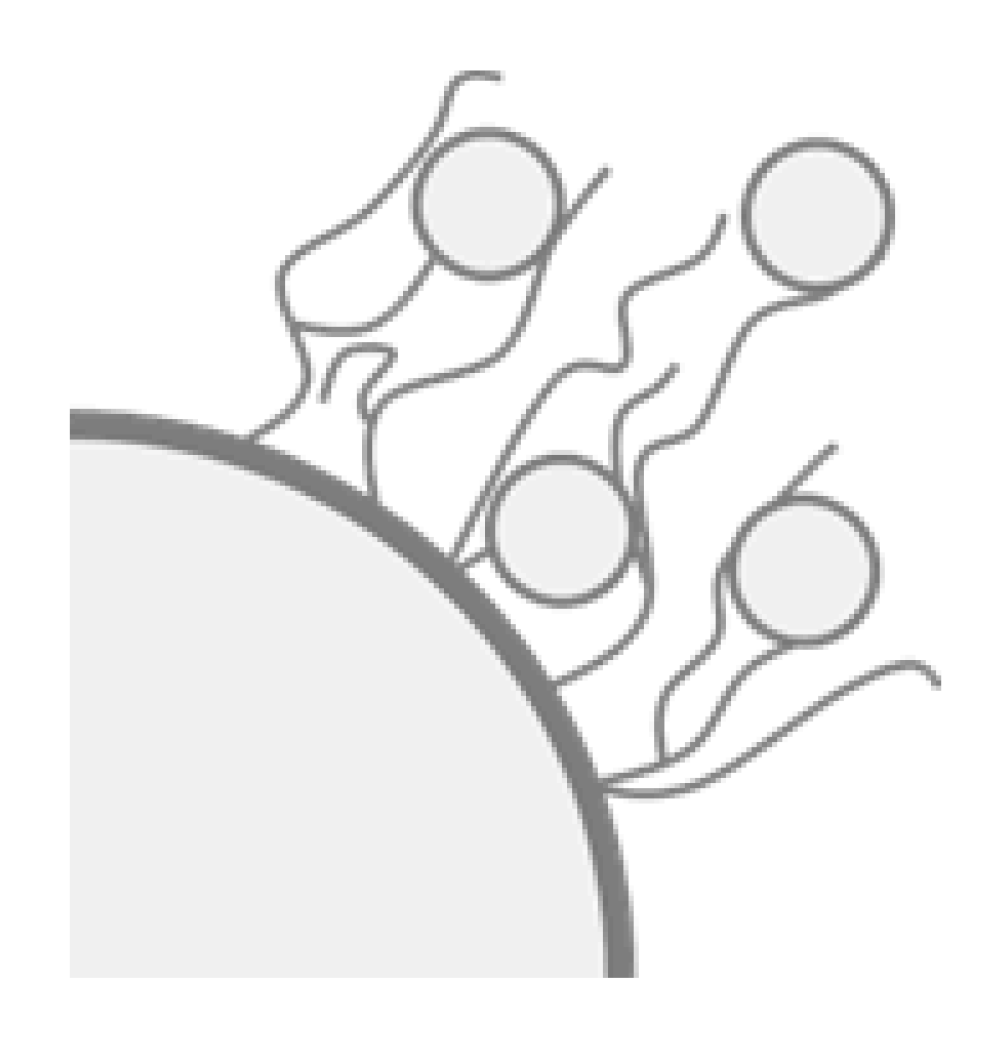
Apoptotic bodies	~50–5000 nm (heterogeneous)	Phosphatidylserine, genomic DNA, caspase 3, histones	Released from cells undergoing apoptosis	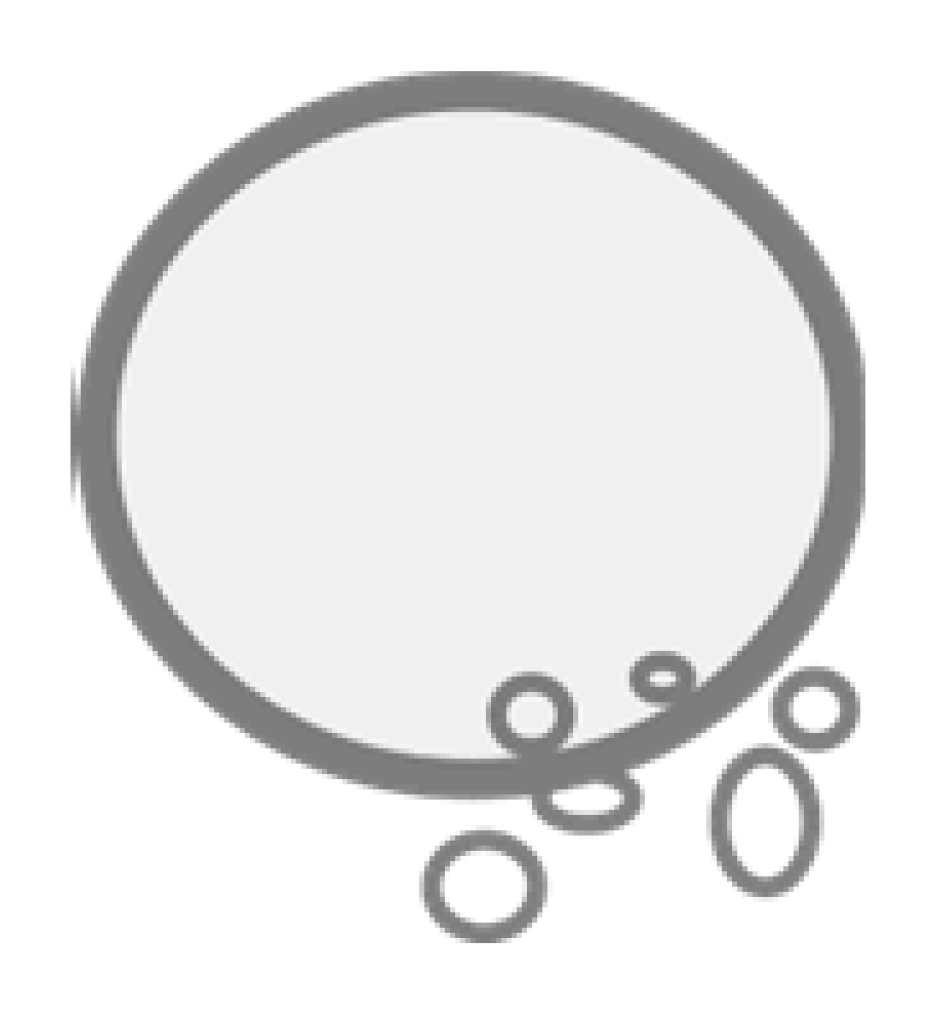

**Table 2:** Common EV *modification* techniques and their specific limitations. Arrows (→) indicate the resulting effect or consequence of the respective method.

Modification Technique	Principle and Limitations
Genetic modification [90,91]	Transfection via viral gene transfer or plasmids (e.g., RUNX2 gene [92] or HIF-1α [93,94]) → high technical complexity, long preparation time
Electroporation [95]	Transfer of exogenous proteins, siRNA, or miRNA by electroporation of the EV membrane → risk of destruction of membrane integrity; potential alterations in the biological properties of producer cells or EVs
Preconditioning through co-cultivation [96]	+ osteogenic induction medium [97,98] induces expression of miRNAs such as let-7a-5p-, let-7c-5p, miR-328a-5p and miR31a-5p + Dimethyloxalylglycine stabilizes HIF-1α [99] + BMP-2 leads to improved bone healing (rats) [100,101,102] + Hypoxia in progenitor cells promotes osteogenesis and angiogenesis [93,103] → Low loading capacity; in some cases, toxic effects of the substances on the source cell or EVs
Sonication [104]	Use of ultrasound → risk of compromising membrane integrity, unstable biological properties of producer cells or EVs
Mechanical extrusion [105,106,107,108]	Mechanical pressing of substances (e.g., using magnetic nanoparticles) → risk of compromising membrane integrity; unstable biological properties of producer cells or EVs
Freezing/thawing [109]	Freeze–thaw cycles → low loading capacity; possible changes in the biological activity of EVs or cargo
Chemical conjugation [110,111]	Covalent bonding of substances → technically complex; loading is limited to specific functional groups

**Table 3:** Representative engineered EV strategies for bone regeneration, including therapeutic cargo, EV source, delivery, bone model, and main therapeutic outcome. i.v.—intravenous. ↑ indicates upregulation, ↓ indicates downregulation.

Therapeutic Cargo	EV Source	Delivery	Bone Model	Main Outcome
Osteogenic/RUNX2 [92]	modified BM-MSCs	In vitro	MSC osteogenesis	↑ Osteogenesis
HIF-1α [94]	HIF-1α-modified BM-MSCs	Local	Rat calvarial defect	↑ Osteogenesis, ↑ angiogenesis
CXCR4/antagomir-188 [91]	NIH-3T3 EV/liposome hybrids	i.v.	Mouse osteoporosis	↑ Bone targeting, ↑ bone mass
BMP-related [101]	BMP-2-overexpressing MSCs	Local	Rat calvarial defect	↑ Osteogenesis, ↑ bone regeneration
TIM3 [100]	TIM3-overexpressing BM-MSCs	Local	Mouse calvarial defect	↓ Inflammation; ↑ bone regeneration
Proangiogenic [99]	DMOG-preconditioned BM-MSCs	Local	Rat calvarial defect	↑ Angiogenesis, ↑ bone formation
Endogenous [102]	BMP-2-stimulated macrophages	Local	MSC osteogenesis	↑ Osteogenic differentiation
miR-1246 [108]	Hypoxia-conditioned iPSC-ECs	i.v.	Mouse osteoporosis	↑ Bone targeting, vascularization and bone formation
Lineage-specific [97]	Osteoblast-/adipocyte-derived EVs	In vitro	MSC differentiation	↑ Lineage-specific differentiation
BMP-2 mRNA [112]	Modified donor cells	Local	Rat calvarial defect	↑ Bone regeneration
VEGF-A/BMP-2 mRNAs [113]	Modified ADSCs	Local	Rat femoral defect	↑ Angiogenesis, ↑ bone regeneration
BMP-2 [114]	Modified donor cells	Local	In vitro/in vivo	↑ Osteogenesis, ↑ BMP-2 stability
BMP-2/BMP-7 [115]	Modified MSCs	Local	Rat calvarial defect	↑ Bone formation, comparable to rhBMP-2

**Table 4:** Common EV isolation techniques, their main principles, and limitations.

Isolation Technique	Main Principle	Main Limitation
Differential ultracentrifugation	Separation by sedimentation rate	Limited specificity; co-isolation of contaminants
Density gradient centrifugation	Separation by particle density	Lower yield; longer processing time
Rate-zonal centrifugation	Separation primarily by sedimentation rate	Gradient-based; technically demanding
Isopycnic centrifugation	Separation by particle density at equilibrium	Relatively time-consuming
Ultrafiltration	Separation according to membrane pore size	Potential EV loss or membrane fouling
Size-exclusion chromatography	Separation according to particle size	Dilution; limited sample capacity
Precipitation	Polymer- or reagent-based EV precipitation	Lower purity; possible reagent contamination
Affinity-based isolation	Capture via specific surface markers	Marker-dependent; potentially limited EV recovery

## Data Availability

No new data were created or analyzed in this study. Data sharing is not applicable to this article.

## Abbreviations

The following abbreviations are used in this manuscript:

ABC	ATP-binding cassette	MMPs	matrix metalloproteinases
ADSCs	adipose-derived stromal cells	MVB	multivesicular body
Akt	protein kinase B	MYD88	myeloid differentiation primary response 88
Ale	alendronate	NF-κB	nuclear factor kappa B
ALP	alkaline phosphatase	OCN	osteocalcin
ARDS	acute respiratory distress syndrome	ONFH	osteonecrosis of the femoral head
AVN	avascular osteonecrosis	PDCD6IP	programmed cell death 6-interacting protein
BM-MSCs	bone marrow-derived mesenchymal stromal cells	PEG	polyethylene glycol
BMP	bone morphogenetic protein	PEGDA	polyethylene glycol diacrylate
CD	cluster of differentiation	PI3K	phosphoinositide 3-kinase
DBCO	dibenzocyclooctyne	PLGA	poly(lactic-co-glycolic acid)
DNA	deoxyribonucleic acid	PRP	platelet-rich plasma
EGFR	epidermal growth factor receptor	RANKL	receptor activator of nuclear factor kappa-B ligand
ESCRT	endosomal sorting complex required for transport	RGD	arginine-glycine-aspartic acid
EV	extracellular vesicle	Ror2	receptor tyrosine kinase-like orphan receptor 2
FasL	Fas ligand	RT	room temperature
HA	hyaluronic acid	RUNX2	runt-related transcription factor 2
HIF	hypoxia-inducible factor	siRNA	small interfering RNA
HIPPO	Hippo signaling pathway	SLC	solute carrier
HSP	heat shock protein	Smad	TGF-β signaling mediator
HUVECs	human umbilical vein endothelial cells	SNARE	soluble N-ethylmaleimide-sensitive factor attachment receptor
ICAM	intercellular adhesion molecule	SSRT	surgical site-released tissue
IL-1β	interleukin-1 beta	TEM	transmission electron microscopy
IL-6	interleukin-6	TEMs	tetraspanin-enriched microdomains
ILV	intraluminal vesicle	TGF-β	transforming growth factor beta
iPS-MSCs	induced pluripotent stem cell-derived mesenchymal stromal cells	TNF-α	tumor necrosis factor alpha
ISEV	International Society for Extracellular Vesicles	TSG101	tumor susceptibility gene 101
lncRNA	long non-coding RNA	TSPAN4	tetraspanin 4
MAPK	mitogen-activated protein kinase	VEGF	vascular endothelial growth factor
MC3T3	mouse osteoblast precursor cell line	Wnt	Wnt signaling pathway
MFGE8	milk fat globule EGF factor 8	β-TCP	beta-tricalcium phosphate
MHC	major histocompatibility complex		
